# Investigating the spatiotemporal expression of *CBTS* genes lead to the discovery of tobacco root as a cembranoid-producing organ

**DOI:** 10.3389/fpls.2024.1341324

**Published:** 2024-05-30

**Authors:** Zaifeng Du, Tian Tian, Yulong Gao, Jian Guan, Fuzhu Ju, Shiquan Bian, Jiahao Wang, Xiaoyang Lin, Bingwu Wang, Zhihua Liao, Yongmei Du, Zhongfeng Zhang, Hongbo Zhang

**Affiliations:** ^1^ Key Laboratory of Synthetic Biology of Ministry of Agriculture and Rural Affairs, Tobacco Research Institute, Chinese Academy of Agricultural Sciences, Qingdao, China; ^2^ Tobacco Breeding and Biotechnology Research Center, Yunnan Academy of Tobacco Agricultural Sciences, Kunming, China; ^3^ School of Life Sciences, Southwest University, Chongqing, China

**Keywords:** *CBTS* genes, tobacco, cembranoid, spatiotemporal expression, root

## Abstract

Tobacco cembranoids, known for their anti-insect and antifungal properties, were shown to be mainly present on the surface of leaves and flowers, being biosynthesized by their trichomes. It remains unclear whether they could be biosynthesized in other organs without trichomes. Cembratrien-ol synthases (CBTSs) catalyze the conversion of GGPP to CBT-ols and thus play an important role in cembranoid biosynthesis. This study identified the *CBTS* family genes in tobacco and examined their spatiotemporal expression patterns. The *CBTS* genes showed diverse expression patterns in tobacco organs, with the majority highly expressed in leaves and a few highly expressed in flowers. The expression of *CBTS* genes were also correlated with the development of tobacco plants, and most of them showed the highest expression level at the budding stage. Furthermore, their expression is mediated by the JA (jasmonate) signaling in all tobacco organs. Several *CBTS* genes were found to be highly expressed in tobacco roots that have no trichomes, which prompted us to determine the cembranoid production in roots and other organs. GC-MS and UPLC assays revealed that cembranoids were produced in all tobacco organs, which was supported by the bioactivity assay results that almost all these CBTS enzymes could catalyze CBT-ol biosyntheis in yeast, and that the content ratio of CBT-ols and CBT-diols in tobacco roots was different to that in leaves. This work sheds insights into the expression profiles of tobacco *CBTS* genes and provides a feasibility to engineer tobacco roots for industrial production of cembranoids.

## Introduction

Cembranoids belong to a class of terpenoid compounds that widely exist in nature (Yan et al., 2016; Huang et al., 2018) and are mainly found in *Nicotiana* spp., pines, and some marine organisms (El Sayed and Sylvester, 2007; Liu et al., 2015; Yang Q. et al., 2020). In *Nicotiana* plants, cembranoids were found principally present in the surface secretion of leaves and flowers. They are the main components of tobacco glandular trichome secretion, accounting for about 60% of the trichome secretion and even more than 0.7% of the fresh leaf weight (Johnson et al., 1985). Generally, CBT-diols are more abundant than CBT-ols in tobacco (Roberts and Rowland, 1962). CBT-ols and CBT-diols both have two isomers that are named as α-/β-CBT-ol and α-/β-CBT-diol (Roberts and Rowland, 1962; Springer et al., 1975). Other forms of cembranoid derivatives have also been identified, and 105 such compounds have been identified in tobacco alone. They can be roughly divided into four groups based on the structural characteristics, that is, cembranoids, nor-cebranoids, seco-cembranoids, and cyclized cembranoids (Wang and Wagner, 2003; Yang P. F. et al., 2020; Xu et al., 2022). CBT-ols and CBT-diols show promising activities in anti-insect (Zhao et al., 2013), antibacterial (Guo and Wagner, 1995; Aqil et al., 2011; Ishii et al., 2016), anticancer (Rodriguez et al., 2010; Nacoulma et al., 2013; Mischko et al., 2018) and neuroprotection (Martins et al., 2015; Velez-Carrasco et al., 2015; Li et al., 2019). An increased cembranoid content did improve plant resistance to aphids and blue mold fungus, and thus CBT-ols might have some potentials as eco-friendly botanical insecticides (Ferchmin et al., 2005; Mischko et al., 2018).

**Figure 1:**
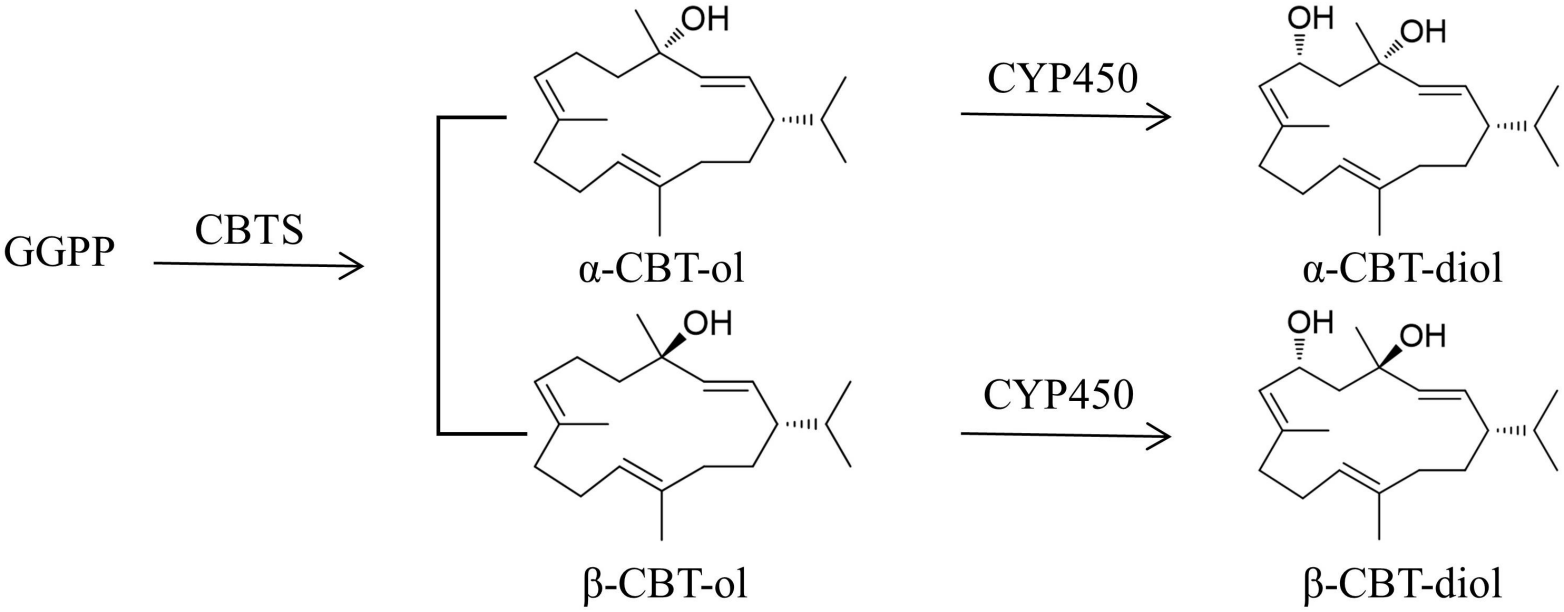
Schematic biosynthetic pathway of CBT-ols and CBT-diols in tobacco. GGPP, geranylgeranyl diphosphate synthase; CBTS, cembratrien-ol synthase; CYP450, cytochrome CYP450 oxygenase.

Cembranoids are the products of terpenoid metabolic pathways, in which isopentenyl diphosphate (IPP) and dimethylallyl diphosphate (DMAPP) are the key precursors (Ma et al., 2006). In plants, IPP and DMAPP could be biosynthesized from acetoacetyl-CoA through the MVA pathway in the cytoplasm or from pyruvate/D-glyceraldehyde-3-phosphate (G3P) through the MEP pathway in the plastids (Tholl et al., 2005; Muhlemann et al., 2014). They are condensed to form geranyl pyrophosphate (GPP) as catalyzed by geranyl diphosphate synthase (GPPS) and then, to GPP, another IPP unit is added by farnesyl diphosphate synthase (FPPS) to form farnesyl diphosphate (FPP) (Cheng et al., 2007). Subsequently, FPP and IPP are converted to geranylgeranyl diphosphate (GGPP), catalyzed by geranylgeranyl diphosphate synthase (GGPPS) (Vranová et al., 2013; Zhang et al., 2020). The GGPPs for cembranoid biosynthesis were supposed to be derived from the MEP pathway (Chappell, 1995; Rohmer, 1999), but recent studies have found that cembranoids could also be biosynthesized through the MVA pathway (Zhang et al., 2020). As illustrated in **Figure 1**, tobacco cembranoids are biosynthesized from the cyclization of GGPP by cembratrien-ol synthase (CBTS) to form α- and β-CBT-ol (Wang and Wagner, 2003; Zhang et al., 2018), and then the sixth carbon of CBT-ols undergo hydroxylation reactions under the action of cytochrome CYP450 oxygenase to generate α- and β-CBT-diol (Wang et al., 2001; Liao et al., 2016).

CBTS is an enzyme with the molecular mass of 58 kDa (Wang et al., 2014) and was shown to mainly exist in glandular trichomes of tobacco leaves (Gershenzon and Croteau, 2018). In 2003, a *CBTS* gene was cloned from *Nicotiana tabacum* and was shown to encode an enzyme catalyzing the formation of CBTS-ols from GGPP (Wang and Wagner, 2003). Studies on the homologous genes *NsCBTS2a* and *NsCBTS2b* demonstrated their main expression in glandular trichome, less in flower and stem, and lowest in roots (Ennajdaoui et al., 2010). Transient expression of the trichome-specific *NsCBTS2a* from *N. sylvestris* could increase the production of CBT-ols (Brückner and Tissier, 2013). Moreover, expression of *NtCBTS2b* or *NtCBTS1* in tobacco can increase the accumulation of CBT-ol in tobacco and enhance tobacco resistance to aphid (Zhang et al., 2018; Guan et al., 2023). These studies suggest an important role of CBTS in the biosynthesis of cembranoids in tobacco.

In view of the multiple activities of cembranoids in medication and pesticide application, their values in pharmacology and sustainable agriculture are attracting more attention than ever. Yet, their yield in tobacco is still insufficient to meet the requirement of industrial production. Thus, it is of great value to metabolically engineer the cembranoid biosynthetic pathway in tobacco to improve their production, or to establish an alternative approach with potential for industrial production. In this study, we cloned the *CBTS* homologous genes in tobacco cultivar TN90 and investigated their spatiotemporal expression characteristics. The relationship between *CBTS* expression and cembranoid production was determined with tobacco organs of different developmental stages. We also determined the expression of certain *CBTS* genes in tobacco roots and revealed tobacco roots as a cembranoid biosynthesizing organ, which might pave the way for the molecular engineering of tobacco roots for an enhanced cembranoid production.

## Materials and methods

### Plant materials and growth conditions

The plants of tobacco (*Nicotiana tabacum* L.) cultivar TN90 were cultivated in an indoor growth room at 25deg;C with a photoperiod of 14 h-light/10 h-dark. The middle leaves of tobacco plants at 5-leaf-stage (S1), 10-leaf-stage (S2), 15-leaf-stage (S3), and budding stage (S4); the upper, middle, and lower leaves; and the flowers and the stems of tobacco plants at flowering stage were collected and frozen in liquid nitrogen for subsequent transcriptional assay and cembranoid quantification, respectively. *NtCOI1*-silenced tobacco plants (COI1-RI) and their control plants (transformed with empty vector) were developed in our previous study (Wang and Liu, 2014).

### Gene sequence analysis

A BLASTP search was performed against the genome data of *N. tabacum* (cv. TN90) at NCBI with the protein sequence of NsCBTS2a (Ennajdaoui et al., 2010) as a query. The obtained homologous proteins are designated as CBTS1–9. Their corresponding genes are under the GenBank accessions XM_016609837.1, XM_016609052.1, XM_016582085.1, XM_016637837.1, XM_016603386.1, XM_016655305.1, XM_016629581.1, XM_016594649.1, and XM_016631693.1, respectively. The gene structure of *CBTS* family genes was analyzed using Gene Structure Display Server 2.0 (GSDS). A phylogenetic tree was constructed with the MEGA version 7.0 software (Kumar et al., 2016) using the deduced protein sequences of NtCBTS1 and other homologous proteins. Alignment assay of the deduced amino acid sequences of the *CBTS* genes was performed using Jalview software (version 2).

### Semi-quantitative RT-PCR and quantitative real-time polymerase chain reaction (qRT-PCR) analysis

Total RNAs of different tobacco samples were extracted with TRIzol™ reagent (Invitrogen, Carlsbad, CA, USA; Cat. No15596026CN), and digested with RNase-free DNase I (Vazyme, Nanjing, China; Cat. NoEN401–01) to remove genomic DNA. The first-strand cDNA was synthesized by reverse transcription using HiScript III All-in-one RT SuperMix Perfect for qPCR kit (Vazyme, Nanjing, China; Cat. NoR333–01) according to the manufacturer’s instruction. Semi-quantitative RT-PCR was amplified with an initial denaturation at 95°C for 2 min and 25 cycles of denaturation at 95°C for 20 s, annealing at 60°C for 20 s, and extension at 72°C for 1 min using gene-specific primers and indicated template cDNAs. *NtActin* gene was amplified in parallel as an internal control. The amplification products of semi-quantitative RT-PCR reactions were separated by electrophoresis in 1.0% (w/v) agarose gel. qRT-PCR was amplified with gene-specific primers and indicated cDNAs using FastStart Universal SYBR Green Master mix (Vazyme, Nanjing, China; Cat. NoQ711–02) according to the manufacturer’s instruction, with *NtActin* gene as an internal control. Specific amplification of target products was accomplished using optimized annealing temperature (60°C–63°C) and monitored by melting curve analysis and sequencing. All qRT-PCR experiments were performed with three independent biological replicates. The relative expression level of each gene was calculated using the 2^-ΔΔCT^ method. Gene specific primers for qRT-PCR and semi-quantitative RT-PCR are listed in **Supplementary Tables S1**, **S2**.

### Extraction of cembranoids from tobacco samples

For quantification of cembranoids in tobacco organs, 1g of freeze-dried samples were extracted with 100 mL of ethyl acetate for three times. An equal volume of deionized water was added to the combined extract and mixed thoroughly to remove the water-soluble substances. After layer separation in a separatory funnel, the cembranoid-containing supernatant was collected and then dried at 40°C in a rotary evaporator under reduced pressure. After dissolving with 10 mL dichloromethane, the extract was collected into a sample bottle and dried in nitrogen flow. Finally, the extract was dissolved in 1 ml of 60% acetonitrile for UPLC (Ultra-Performance Liquid Chromatography) assay, or dissolved in 1 ml of ethyl acetate for GC-MS (Gas Chromatograph-Mass Spectrometry) assay.

### Quantification of cembranoids by UPLC assay

For quantification of cembranoids by the aid of UPLC, the tobacco extract dissolved in 60% acetonitrile was filtered through a 0.22-μm pore size filter and injected into a UPLC machine (Waters Technologies, USA) equipped with a BEH C18 column (1.7 µm, 2.1 mm × 100 mm) under the following conditions: the column temperature of 40°C, a gradient mobile phase as described by Zhang et al. (2020) at the flow rate of 0.3 mL/min, and a UV detector for detection of CBT-ol and CBT-diol at 208 nm. The authentic standards of CBT-ol and CBT-diol, which were isolated and purified from a 95% EtOH extract of tobacco trichomes using a preparative HPLC system as previously described (Zhang et al., 2020), were used to plot the standard curves for quantification of α-/β-CBT-ol and α-/β-CBT-diol.

### Identification of cembranoids by GC-MS assay

For identification of cembranoids by GC-MS assay, the tobacco extract dissolved in ethyl acetate was filtered through a 0.22 μm pore size filter and injected into a GC-MS machine (Agilent, USA) equipped with capillary column HP-5MS (30 m × 250μm × 0.25μm) under following optimized parameters: the initial column temperature was set at 80°C and maintained for 1 min, increased to 200°C at 15°C/min and maintained for 1 min, and then raised to 240°C at the rate of 4°C/min and maintained for 2 min. The mass spectra were obtained at m/z 50–650 using negative ionization mode at 70 eV (EI). The authentic standards of CBT-ol and CBT-diol were used as reference. The peaks for CBT-ol and CBT-diol were identified by retention time comparison with the reference standards and mass spectra matching against the NIST Database.

### Determination of the bioactivity of CBTS members in yeast

The cDNA fragment of each *CBTS* gene was amplified using 2×Phanta^®^ Max Master (Vazyme, Nanjing, China; Cat. NoPP525–01) with gene specific primers (**Supplementary Table S3**) and then cloned into the vector pGADT7 (Clontech, USA) by In-Fusion^®^ (Takara, Shiga, Japan; Cat. No639649) cloning method for expressing the corresponding enzyme in yeast. The derived vectors were introduced into the yeast strain BY-T20 (*MATα, trp1Δ0, leu2Δ0, ura3Δ0, trp1::HIS3-P_PGK1_-BTS1/ERG20-T_ADH1_-P_TDH3_-SaGGPS-T_TPI1_-P_TEF1_- tHMG1-T_CYC1_*) (Hu et al., 2020). The yeast transformants were cultivated on SD/-Leu medium (Takara, Shiga, Japan; Cat. No630311) to obtain the desired positive colonies. The obtained positive colonies of each CBTS enzyme were inoculated into 10 mL of liquid SD/-Leu medium and cultured at 30deg;C and 220 r/min for 48h to be used as seed culture. The seed culture was inoculated into YPD liquid medium (2% glucose, 1% yeast extract, 2% peptone) in a flask at an inoculation ratio of 20% (v/v), and cultured at 30°C and 220 r/min for 72h. For cembranoid extraction, 1 L of the cell culture was collected and centrifuged to separate yeast cells from the cultivation broth. The yeast cells were weighed and grounded into fine powder in liquid nitrogen, and then lysed in 20 mL ddH_2_O using an ultrasonic cell disruptor for 20 min. The cell lysate was extracted three times with 100 mL of ethyl acetate for 30 min at 30deg;C with agitation. After centrifugation, the upper organic phase of the extract was collected and combined for further concentration. The combined extract was dried in a rotary evaporator at 40deg;C, and dissolved in 5 mL of ethyl acetate for further GC-MS or UPLC assay as described above.

### Statistical analysis

Statistical analyses of the quantitative data were performed using Microsoft Excel. Significance analysis and correlation analysis were conducted with the SPSS software (version 26.0) and analyzed by one-way analysis of variance with Dunnett’s test at *P* < 0.05. The histograms were drawn using Origin software (version 2021).

## Results

### Sequence analysis of *CBTS* family genes

**Figure 2:**
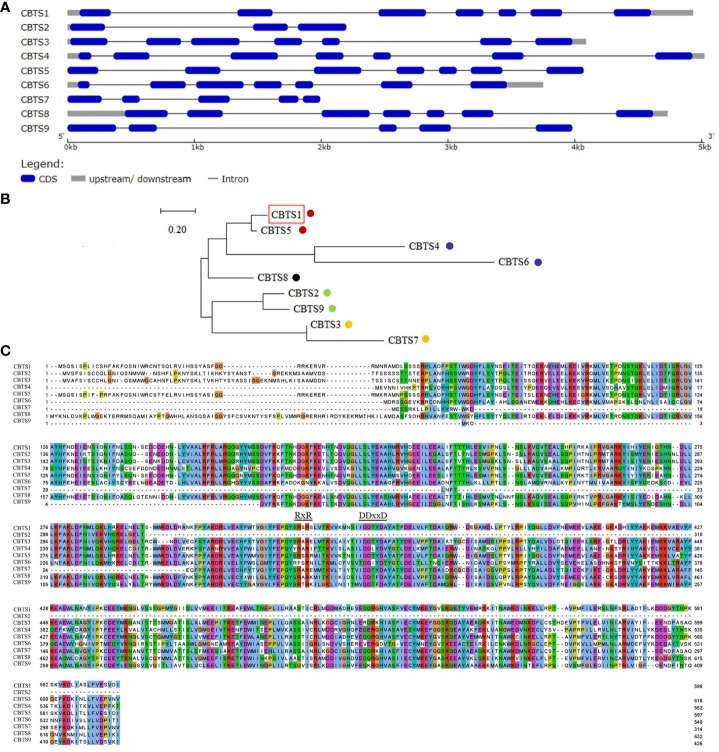
Sequence analysis of *CBTS* family genes. **(A)** Schematic diagram of the gene structures of *CBTS* genes. **(B)** The phylogenetic analysis of *CBTS* genes. The CBTS-a, CBTS-b, CBTS-c, CBTS-d, and CBTS-e subgroups are marked with red, purple, black, green, and orange dots, respectively. Red boxes indicate that the corresponding genes were previously studies. **(C)** Alignment of the deduced amino acid sequences of *CBTS* genes. Colors indicate different types of amino acids. The DDxxD and the RxR domains are marked with letters and underscores, respectively.

By searching the accessible genomic data at GenBank of NCBI, nine homologs of NsCBTS2a (Ennajdaoui et al., 2010) were identified in the genome of *N. tabacum* cv. TN90. One of them is CBTS1 that showed activity in catalyzing cembranoid biosynthesis (Zhang et al., 2020), and the other eight proteins were designated as CBTS2–9. Sequence analysis of the corresponding *CBTS* genes showed that the mRNA sequences of *CBTS* genes have a high similarity (up to 96%) to each other (**Figure 2**). The gene structural analysis showed that *CBTS8* has the longest mRNA sequence and that *CBTS2* has the shortest one (**Figures 2A, C**). The number of exons in the *CBTS* genes ranged from three to seven, and the majority of them contained seven exons (**Figure 2A**). To dissect the evolutionary relationship of these *CBTS* genes, sequence alignment, and phylogenetic analysis were performed based on their deduced amino acid sequence. As shown in **Figure 2B**, these CBTSs could be divided into five subgroups, that is, CBTS-a, CBTS-b, CBTS-c, CBTS-d, and CBTS-e.

There are two conserved domains, that is,”DDxxD” and “RxR” domains, located at the C terminals of the deduced CBTS proteins (**Figure 2C**). The DDxxD domain endows CBTS enzymes the Class I activity of terpene synthase (TPS) to directly catalyze the biosynthesis of diterpenes from GGPP (Liu et al., 2017). The RxR domain prevents nucleophilic attack on the carbocation intermediate (Christianson, 2017). Yet, the CBTS2 encodes a terpenoid synthase without the DDxxD motif.

### Organ-specific expression profiles of *CBTS* genes

**Figure 3:**
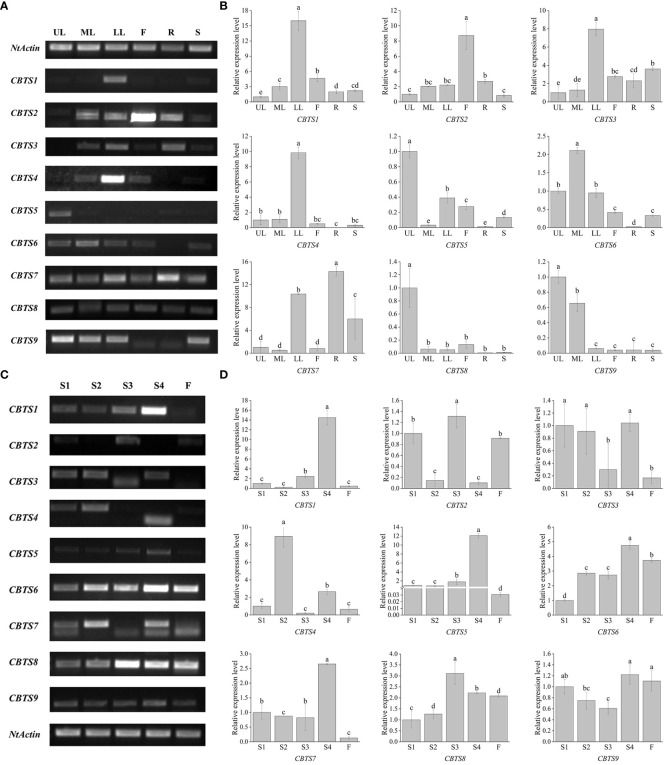
Expression of *CBTS* genes in different organs of tobacco plants. **(A, B)** Semi-quantitative real-time polymerase chain reaction (RT-PCR) **(A)** and qRT-PCR **(B)** analyses of *CBTS* gene expression at flowering stage. UL, ML, and LL indicate the upper, middle, and lower leaves of plants at flowering stage, F indicates flower, R indicates root, and S indicates stem. **(C, D)** Semi-quantitative RT-PCR **(C)** and qRT-PCR **(D)** analyses of *CBTS* gene expression in the leaves at different development stages. Tobacco growth stages are indicated as S1 (5-leaf-stage), S2 (10-leaf-stage), S3 (15-leaf-stage), S4 (budding stage), and F (flowering stage). Genes having alternative splicing are marked with asterisks in **(C)**. For qRT-PCR assays, the shown values are means ± SD (*n* = 3). The expression of each gene in UL **(C)** and in leaves at S1 **(D)** was arbitrarily set as “1”. Lowercase letters indicate significant differences from control plants (p<0.05).

The organ-specific expression of *CBTS* genes was determined by semi-quantitative RT-PCR and qRT-PCR assays, using cDNAs from different organs of tobacco plants at the flowering stage. The semi-quantitative RT-PCR assay showed that most of the *CBTS* genes exhibited organ-specific expression patterns, except for *CBTS8* that was expressed at similar levels in all the tested organs (**Figure 3A**). The qRT-PCR data for *CBTS* genes are consistent with the semi-quantitative RT-PCR results (**Figure 3B**). *CBTS1/3/4/7* were expressed at much higher levels (up to sixfold) in the lower leaves than in the upper leaves, while *CBTS5/8/9* were expressed at the highest levels (twofold to 15-fold) in the upper leaves, as compared to that in the lower leaves (**Figure 3A**). The transcripts of *CBTS2* were most abundant in the flowers and those of *CBTS6* was most abundant in the middle leaves (**Figures 3A, B**). The transcripts of *CBTS2* were most abundant in the flowers, while those of *CBTS6* were most abundant in the middle leaves (**Figures 3A, B**). Furthermore, both *CBTS2* and *CBTS6* were expressed at certain levels in the leaves of different positions (**Figures 3A, B**), which suggested that the majority of the *CBTS* genes are expressed in leaves. In addition, *CBTS5/8* showed a considerable expression in flowers, and *CBTS5/6/7/9* some notable expression in stems (**Figures 3A, B**). Strikingly, *CBTS2/3/7* were expressed at a certain level in roots, and *CBTS7* showed the highest level in roots (**Figures 3A, B**), which implied the potentials of tobacco roots in biosynthesizing cembranoids.

The expression of *CBTS* genes in the leaves of tobacco plants at different developmental stages was also determined. The semi-quantitative RT-PCR showed that most of the *CBTS* genes had a high expression level in the leaves of tobacco plants at S4 stage (**Figures 3C, D**). *CBTS1/7* showed high-expression levels in the leaves of tobacco at S4 stage but were less expressed in the tobacco at flowering stage (**Figures 3A, C**). The qRT-PCR results showed similar gene expression trends as the semi-quantitative RT-PCR. *CBTS1*/3/*5/6/7/9* are mainly expressed in the leaves of tobacco at S4 stage, which were 1.1- to 14-folds of their expression at other stages, while *CBTS2/3/4/8* were observed to be highly expressed during S1-S3 stages (**Figure 3D**). The majority of the *CBTS* genes showed decreased expression levels in the leaves at flowering stage (**Figure 3D**). Above data suggested that most of *CBTS* genes are highly expressed at S4 stage, that is, the budding stage that is closely preceding the flowering stage. Interestingly, alternative splicing of *CBTS* gene transcripts (e.g., *CBTS3*/*4*/*7*) was observed in the semi-quantitative RT-PCR assays (**Figure 3C**), which showed different splicing patterns in the leaves at different developmental stages.

### Regulation of *CBTS* gene expression by jasmonate (JA) signaling

**Figure 4:**
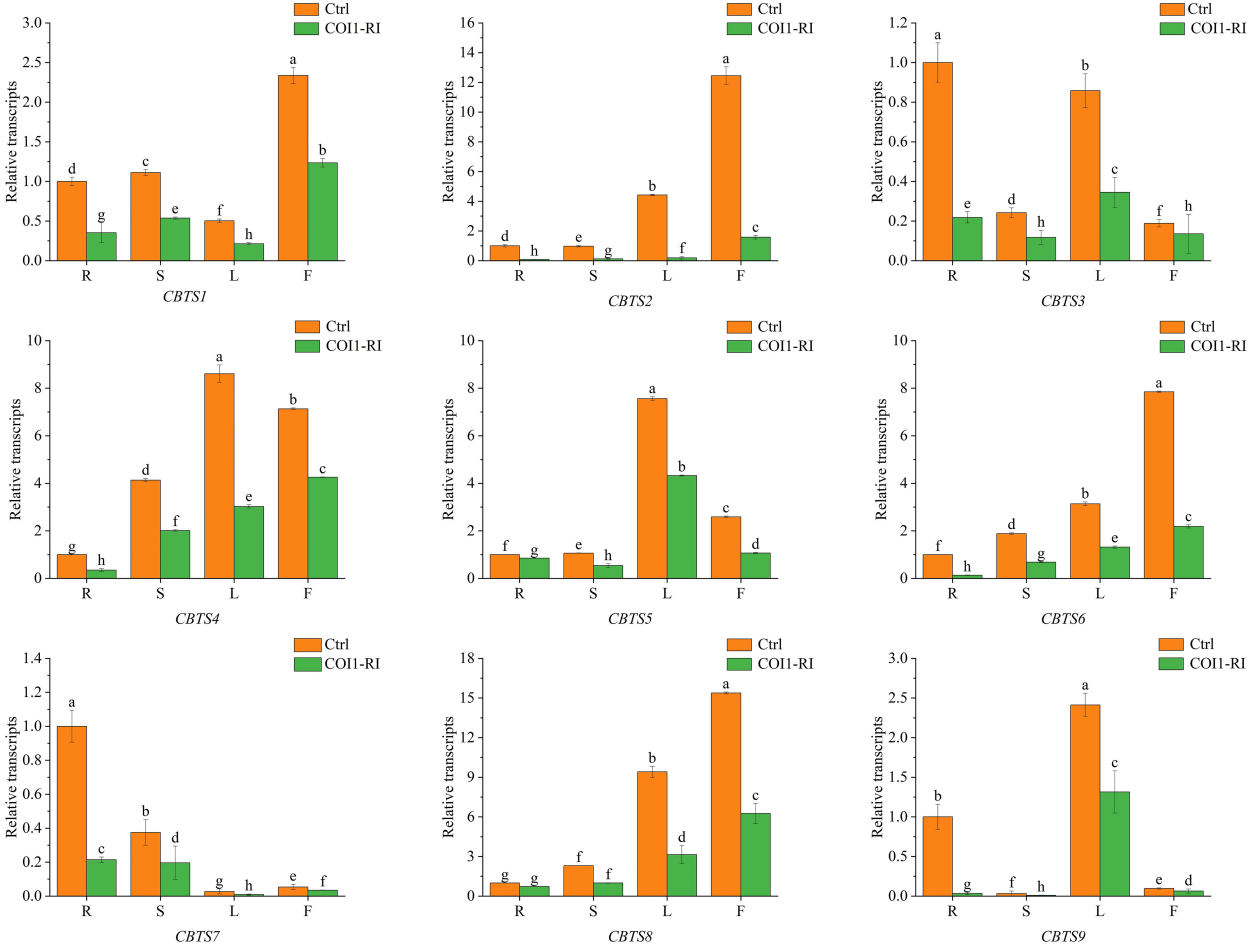
Expression of *CBTS* genes in different organs of tobacco plants with dysfunction of COI1. Ctrl indicates control plants transformed with empty vector, and COI1-RI indicates plants with dysfunction of COI1. R indicates root, S indicates stem, L indicates leaf, and F indicates flower. The shown values are means ± SD (*n* = 3). The expression of each gene in the root of control plants was arbitrarily set as “1”. Lowercase letters indicate significant differences from control plants (p<0.05).

Previous studies showed that the biosynthesis of cembranoids in tobacco is regulated by JA (Sui et al., 2018). In order to determine the JA-mediated expression of *CBTS* genes, their expression in the tobacco plants with dysfunction of the JA receptor protein COI1 (COI1-RI) were analyzed. The transcriptional analyses showed that the expression of *CBTS* genes in COI1-RI plants was significantly lower than that in control plants (transformed with empty vector). The expression of *CBTS1*/2/3/*4/6/7/9* in the root of COI1-RI plants decreased by over 60% compared with control plants, while the expression of *CBTS5/8* in the root of COI1-RI plants reduced about 20% (**Figure 4**). The expression of all the *CBTS* genes were attenuated by around 50% in the stems and leaves of COI1-RI plants compared with control plants (**Figure 4**). The expression of *CBTS1/2/5/6/8* in the flowers of COI1-RI plants decreased by over 50% compared with control plants, the expression of *CBTS4* in the flowers of COI1-RI plants decreased about 40%, and the expression of *CBTS3/7/9* in the flowers of COI1-RI plants decreased around 20% (**Figure 4**). These results demonstrated that the expression of *CBTS* family genes in tobacco is regulated the JA-signaling pathway.

### Tobacco root is a cembranoid-producing organ

**Figure 5:**
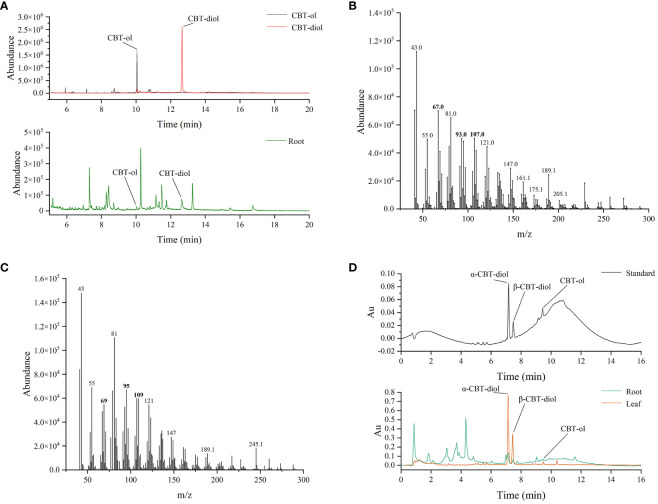
Identification and quantification of cembranoids in tobacco roots. **(A)** GC-MS spectrum of CBT-ol and CBT-diol in the extract of tobacco roots. **(B, C)** The associated mass peaks of CBT-ol **(B)** and CBT-diol **(C)**. Bold number indicate the mass peaks discriminating CBT-ol from CBT-diol. **(D)** UPLC analysis of CBT-ols and CBT-diols in the extract of roots and leaves.

**Figure 6:**
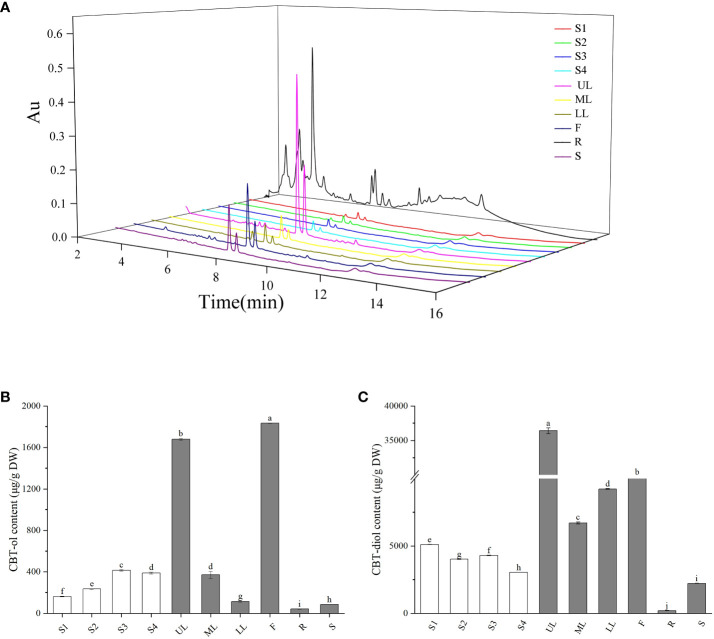
Accumulation of cembranoids in different tobacco organs. **(A)** UPLC spectrum of cembranoids in different organs. **(B, C)** CBT-ols **(B)** and CBT-diols **(C)** contents in different organs. S1–S4 indicate the middle leaves of tobacco plants at 5-leaf-stage, 10-leaf-stage, 15-leaf-stage, and budding stage, respectively. UL, ML and LL indicate the upper, middle and lower leaves of tobacco plants at flowering stage, respectively. F, R, and S indicate the flowers, roots and stems of tobacco plants at flowering stage, respectively. The values shown in B and C are means ± SD (*n* = 3).

Previous studies suggested that tobacco cembranoids mainly exist in the secretion of glandular trichomes (Johnson et al., 1985). The above findings showed that several *CBTS* genes such as *CBTS3* and *CBTS7* were expressed at certain levels in tobacco roots (**Figure 3**), and we were thus prompted to investigate the role of tobacco roots in cembranoid biosynthesis. The freeze-dried tobacco root samples were ground into powder, extracted with ethyl acetate, and then subjected to cembranoid analysis using GC–MS assay. The GC–MS analysis of extracts from tobacco roots led to identify two ion peaks corresponding to CBT-ol and CBT-diol, with retention times of 10 min and 13 min, respectively (**Figure 5A**). A set of characteristic peaks for CBT-ol and CBT-diol were observed in the GC–MS data, and the peaks for distinguishing CBT-ol and CBT-diol were also detected (**Figures 5B, C**). In the UPLC analysis, three peaks consistent with the retention times of α-CBT-diol, β-CBT-diol, and CBT-ols standards (α-CBT-ol and β-CBT-ol were not separated in UPLC) were detected in the sample from tobacco roots (**Figure 5D**). These results suggested that tobacco roots have the capability to produce CBT-ols and CBT-diols. To quantify the cembranoid production in tobacco roots, standard curves for UPLC assay were plotted with a serial dilution of CBT-ols and CBT-diols, which were isolated and purified from tobacco leaf trichomes (Zhang et al., 2020). **Figure 5D** showed the UPLC spectra of cembranoid standards and the extract from roots and leaves. Apparently, the contents of α-CBT-diol and β-CBT-diol in tobacco roots were 107.55 μg/g and 33.43 μg/g, respectively, while that of CBT-ols was 17.69 μg/g (**Figures 5D**, **6**). In contrast, the contents of α-CBT-diol and β-CBT-diol in tobacco leaves were 2577.59 μg/g and 756.30 μg/g, respectively, and that of CBT-ol was 103.54 μg/g (**Figures 5D**, **6**). Therefore, the content ratio of α-CBT-diol/β-CBT-diol was about 3.2:1.0 in tobacco roots and about 3.4:1.0 in tobacco leaves, and the content ratio of CBT-ols/CBT-diols was about 8.0:1.0 in tobacco roots and about 32.2:1.0 in tobacco leaves. These findings showed that tobacco roots and leaves had a similar biosynthetic pattern for α-CBT-diol and β-CBT-diol, but a different biosynthetic pattern for CBT-ols and CBT-diols.

### Characterization of cembranoid accumulation in tobacco organs

Cembranoid accumulation in the middle leaves of tobacco plants at seedling stage (S1–S3), budding stage (S4), and flowering stage was analyzed by UPLC analysis (**Figure 6A**). During the seedling stages, the CBT-ols content in tobacco leaves was around 162.58 µg/g at S1 and gradually increased to over 415.14 µg/g at S3, and the CBT-diols content was 5115.20 µg/g at S1 and decreased to a slight lower level at S3 (**Figures 6B, C**), which showed different accumulation trends for CBT-ols and CBT-diols in the middle leave of tobacco plants. At S4, both CBT-ols and CBT-diols showed a slight decrease compared with that at S3 (**Figures 6B, C**).

The accumulation of cembranoids in different organs was analyzed with tobacco plants at the flowering stage. The results showed that CBT-ols were most abundant in the flowers with a content of 1834 µg/g, while CBT-diols were most abundant in the upper leaves with a content of 36420 µg/g (**Figure 6B**). CBT-ols and CBT-diols were both detected in the roots and stems, but were found at lower levels (**Figures 6B, C**). The content of CBT-ols was less than 100 µg/g in both the roots and stems. The content of CBT-diols was less than 300 µg/g in the roots, but over 2000 µg/g in the stems (**Figures 6B, C**).

### Analysis of the bioactivity of tobacco CBTSs in yeast

**Figure 7:**
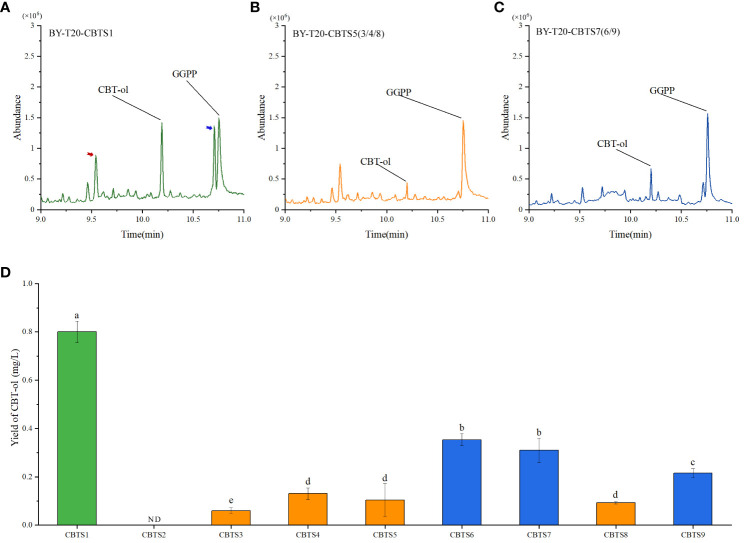
Production of CBT-ol in yeast strain BY-T20 expressing CBTS family genes. **(A–C)** Representative GC–MS spectra of yeast extract expressing indicated CBTS enzymes. The GC–MS peaks for CBT-ol and GGPP are indicated. Red and blue arrows in **(A)** indicate Cyclo (Leu-Pro) and Cyclo (Leu-Leu) detected in the extract, respectively (MS spectrum data shown in **Supplementary Figure S1**). **(D)** Content of CBT-ol in the yeast culture expressing indicated CBTS enzyme. ND indicates that CBT-ol was not detectable. Each value is the average of triplicates and is shown as mean ± SD.

CBTS enzyme catalyzes the biosynthesis of CBT-ol from GGPP, and it can catalyze the biosynthesize of CBT-ol in *Saccharomyces cerevisiae* (Zhang et al., 2020). This work further introduced the obtained *CBTS* genes, respectively, into the yeast strain BY-T20 for a heterogeneous expression to determine the bioactivity of their encoding enzymes in catalyzing the biosynthesis of CBT-ol. The production of CBT-ol for each enzyme was measured with yeast cells from 1 L of yeast culture. The results showed that these enzymes could by classified into three groups based on bioactivity, and their representative GC–MS spectra were shown in **Figures 7A–C**. The corresponding quantification results of UPLC assay turned out that CBT-ol content in the yeast culture expressing CBTS1 was near 0.8 mg/L, in that expressing CBTS6, CBTS7, or CBTS9 was about 0.2–0.4 mg/L, and in those expressing other CBTS enzymes were lower than 0.2 mg/L (**Figure 7D**). Above sequence analysis classified the CBTSs into five groups including CBTS-a, CBTS-b, CBTS-c, CBTS-d, and CBTS-e (**Figure 2B**), yet low bioactivity enzymes (i.e., CBTS2/3/4/5/8) were presented in all these groups and high bioactivity enzyme (i.e., CBTS1) was only presented in the CBTS-a group (**Figure 7D**). Among these enzymes, only CBTS2 which is the shortest one in sequence (**Figure 2**) showed no bioactivity in catalyzing the formation of CBT-ol (**Figure 7D**). Taken together, these results suggested that the CBTS enzymes might function in most of the tobacco organs depending their expression levels, and also supported tobacco root as a cembranoid-producing organ.

## Discussion

Tobacco is abundant in the production of cembranoids that have antifungal and anti-insect activities and great potentials for developing eco-friendly agricultural chemicals (Wang et al., 2001; Mischko et al., 2018). Cembratrien-ol synthase (CBTS) catalyzes the conversion of GGPP to CBT-ols and plays an important role in the biosynthesis of cembranoids (Reid, 1974). In this study, we identified the *CBTS* family genes in tobacco cultivar TN90, analyzed their sequence characteristics and spatiotemporal expression patterns, and investigated the correlation between *CBTS* expression and cembranoid accumulation. Findings in study evidenced that the expression of *CBTS* genes and the biosynthesis of cembranoids could happen in organs other than the trichomes of tobacco leaves and flowers.

This work examined the expression patterns of *CBTS* family genes in tobacco, and found that the *CBTS* family genes were expressed differentially in tobacco organs. Most of the *CBTS* genes had relative higher expression levels in the upper, middle or lower leaves of tobacco plants at flowering stages, *CBTS2* was expressed at the highest level in the flowers and roots and *CBTS6*/*8* mainly expressed in leaves at budding stage. Moreover, the expression levels of all the *CBTS* genes were decreased in the tobacco plants with dysfunction of COI1, the receptor protein of JA, as previous findings on *CBTS1* (Sui et al., 2018). These findings indicated a critical role of JA-signaling in modulating the expression of *CBTS* genes in tobacco. Strikingly, *CBTS7* were found to be highly expressed in tobacco roots and *CBTS2/3* also showed relative higher expression levels the roots. The expression of *CBTS* genes in versatile organs of tobacco plant implied that CBTS enzymes may catalyze the cembranoid biosynthesis in tobacco organ other than leaves and flowers. Their expression in roots prompted us to speculate that cembranoids may also be biosynthesized in tobacco roots. The study with GC-MS and UPLC assays identified both of CBT-ol and CBT-diol in the extract of tobacco roots, which proved that tobacco root is an organ capable of biosynthesizing cembranoids. Further cembranoid quantification with UPLC revealed that the content ratio of α-CBT-diol/β-CBT-diol in tobacco roots was similar to that in tobacco leaves. It was also close to the previously observed content ratio of α-CBT-diol/β-CBT-diol (2:1–3:1) in tobacco leaves or flowers (Shi and Liu, 1998). However, the content ratio of CBT-ols/CBT-diols in tobacco roots was about 8.0:1.0, and it was about 32.2:1.0 in tobacco leaves. These findings evidenced tobacco root as a cembranoid-producing organ for the first time, and implied that the cembranoid biosynthetic patterns in tobacco roots may differ from that in tobacco leaves and flowers.

In previous studies, less attention was paid to the dynamics of cembranoid accumulation and *CBTS* gene expression in different tobacco organs (Ennajdaoui et al., 2010; Zhang et al., 2018). When analyzing the spatiotemporal expression patterns of *CBTS* genes, we also determined the cembranoid content in tobacco organs at different developmental stages. The findings showed that the accumulation patterns of cembranoids were significantly different in tobacco organs, and it was greatly affected by the developmental stages of tobacco plants. Approximately, the accumulation characteristics of CBT-diols were consistent with that of CBT-ol, and both of them were highly accumulated in the flowers and leaves of plants at flowering stage, especially in the upper leaves. This accumulation trend was supported by the expression pattern of *CBTS* genes (**Table 1**), which showed that several *CBTS* genes including *CBTS5/8/9* were expressed at relatively higher levels in the upper leaves of tobacco plants at flowering stage and that *CBTS2* was specifically expressed in the flowers. The difference of cembranoid content and gene expression in leaves at the same developmental stage but from different positions showed a development-stage dependence of cembranoid biosynthesis. Whereas, the leaf CBT-diols of tobacco plants at seedling stages showed an opposite accumulation trend compared with that of CBT-ols. This phenomenon should be correlated with the conversion of CBT-ol to CBT-diol during the seedling stage of tobacco plants and indicated a complicated regulation of the biosynthesis of cembranoids in tobacco. Further bioactivity assay in yeast revealed that all these CBTS enzymes except for CBTS2 showed different bioactivities in catalyzing the biosynthesis of CBT-ol, and that CBTS1 had the highest bioactivty while CBTS6/7 had a medium bioactivity. This evidence supported tobacco root as a cembranoid-producing organ and also suggested that the CBTS enzymes might function in most of the tobacco organs.

**Table 1:** Correlation analysis between cembranoid content and the expression level of *CBTS* genes.

Component	CBTS1	CBTS2	CBTS3	CBTS4	CBTS5	CBTS6	CBTS7	CBTS8	CBTS9
CBT - ol	-0.268	0.545*	-0.412	-0.289	0.633**	0.010	-0.673**	0.638**	0.421
CBT - diol	-0.227	0.219	-0.373	-0.149	0.866**	0.128	-0.657**	0.856**	0.652**

Asterisks indicate significant difference (*P < 0.05; **P < 0.01).

Nevertheless, the semi-quantitative RT-PCR results showed the presence of alternative splicing of the transcripts of *CBTS* family genes, and their splicing patterns are correlated with the developmental stages of tobacco plants. Alternative splicing of gene transcripts exists widely in plants and is one of an important way for plant to regulate the diversity of gene functions (Feng et al., 2020). The alternative splicing of *CBTS* gene transcripts mainly occurred during the seedling stages of tobacco, when the accumulation of cembranoids was at relatively lower levels. Presumably, alternative splicing is a possibility for tobacco plants to suppress the function of CBTS enzymes at the seedling stages. These findings indicated that the function of *CBTS* genes in tobacco plants can be regulated at multiple levels.

Taken together, this study identified the *CBTS* family genes in tobacco and revealed their spatiotemporal expression patterns in the organs of different developmental stages. The finding of root-specific expression of *CBTS* genes lead to the identification of tobacco root as a cembranoid-producing organ, which was also supported by the bioactivity assay results. These findings provide insights into the biosynthesis of cembranoids in tobacco and alternative approach for metabolic engineering of tobacco roots for industrial production of cembranoids.

## Data availability statement

The original contributions presented in the study are included in the article/**Supplementary Material**. Further inquiries can be directed to the corresponding author.

## Author contributions

ZD: Writing – original draft, Data curation, Conceptualization, Formal analysis, Investigation, Methodology. TT: Data curation, Investigation, Methodology, Writing – original draft. YG: Data curation, Formal analysis, Investigation, Methodology, Validation, Writing – original draft. JG: Data curation, Formal analysis, Investigation, Methodology, Validation, Writing – original draft. FJ: Data curation, Formal analysis, Investigation, Validation, Writing – original draft. SB: Data curation, Formal analysis, Investigation, Methodology, Validation, Writing – original draft. JW: Data curation, Formal analysis, Investigation, Methodology, Validation, Writing – original draft. XL: Data curation, Formal analysis, Investigation, Methodology, Writing – original draft. BW: Writing – review & editing, Formal analysis, Methodology. ZL: Writing – review & editing, Methodology, Supervision. YD: Writing – review & editing, Formal analysis, Validation. ZZ: Data curation, Writing – review & editing, Formal analysis, Funding acquisition, Validation. HZ: Writing – review & editing, Writing – original draft, Conceptualization, Funding acquisition, Supervision, Validation.
