# Supplementary material for: Investigating the spatiotemporal expression of CBTS genes lead to the discovery of tobacco root as a cembranoid-producing organ
Source: Front Plant Sci. 2024 May 30;15:1341324. doi: 10.3389/fpls.2024.1341324 (PMC11169922; doi:10.3389/fpls.2024.1341324)
Supplement: Supplementary file 1 [file DataSheet_1.docx]

***Supplementary Material***

**Investigating the spatiotemporal expression of *CBTS* genes lead to the discovery of tobacco root as a** **cembranoid****-producing organ**

Zaifeng Du^1^, Tian Tian^1^, Yulong Gao^2^, Jian Guan^1^, Fuzhu Ju^1^, Shiquan Bian^1^, Jiahao Wang^1^, Xiaoyang Lin^1^, Bingwu Wang^2^, Zhihua Liao^3^, Yongmei Du^1^, Zhongfeng Zhang^1^, and Hongbo Zhang^1,^*

^1^Key Laboratory of Synthetic Biology of Ministry of Agriculture and Rural Affairs, Tobacco Research Institute, Chinese Academy of Agricultural Sciences, Qingdao 266101, China;

^2^Tobacco Breeding and Biotechnology Research Center, Yunnan Academy of Tobacco Agricultural Sciences, Kunming 650021, China;

^3^School of Life Sciences, Southwest University, Chongqing 400715.

^*^Correspondence: Corresponding Author: E-mail: [zhanghongbo@caas.cn](mailto:zhanghongbo@caas.cn).

**Supplementary Figure**

**
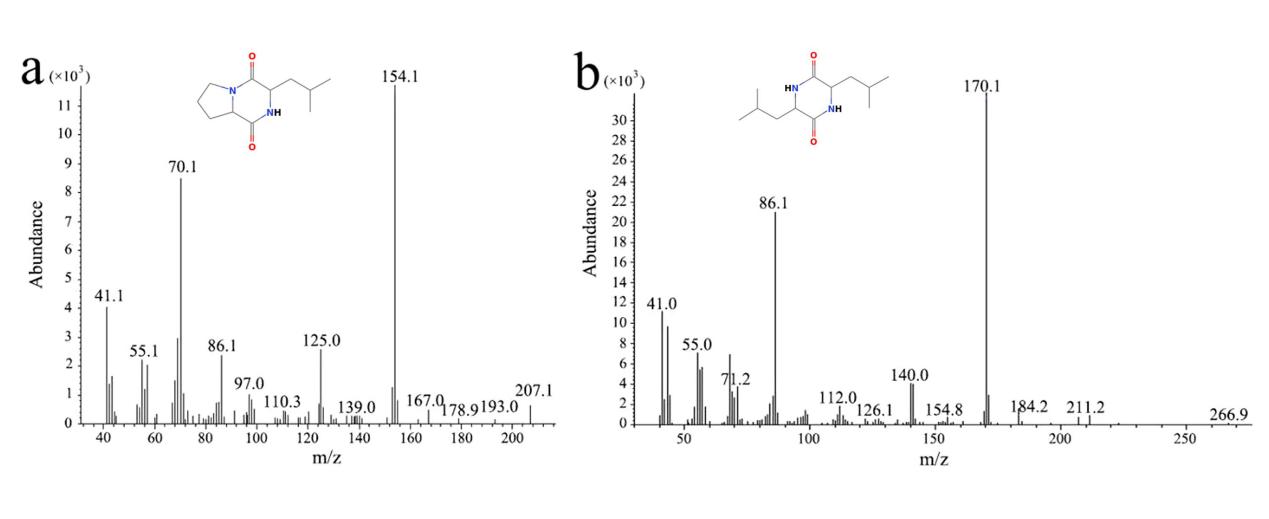
**

**Figure S1.** MS spectrum of Cyclo (Leu-Pro) and Cyclo (Leu-Leu) detected in the indicated yeast extract in Figure 7. a, Cyclo (Leu-Pro); b, Cyclo (Leu-Leu).

**Supplementary Tables**

**Table S1.** Primers for qRT-PCR

| **Gene** | **NCBI Accession** | **Primer Forward(5’-3’)** | **Primer Reverse(****5’-3’)** |
| --- | --- | --- | --- |
| *NtActin* | [CAA45149.1](https://www.ncbi.nlm.nih.gov/protein/22609) | CCACACAGGTGTGATGGTTG | GTGGCTAACACCATCACCAG |
| *CBTS1* | XM_016609836.1 | ACGAGCTAACAAGCTGGTGG | CGCATCCGTGAAAAGCACAA |
| *CBTS2* | XM_016609052.1 | TCTTACACTCCTCAACTCACAGAA | ATGCCACTCCCAATCGTTGAAA |
| *CBTS3* | [XM_016582085.1](https://www.ncbi.nlm.nih.gov/nuccore/XM_016582085.1) | CCAACGTTTGTCCTCGAACT | TCTCAAACTACATTATTTTGCTCCA |
| *CBTS4* | XM_016637837.1 | TCCAAGTGTTTGGGGTGACT | CGATACTCCCAGTCGTTGGA |
| *CBTS5* | [XM_016603386.1](https://www.ncbi.nlm.nih.gov/nuccore/XM_016603386.1) | CTTGCGAGGCAACAAGGAAA | TGGACTCGAGATGAGTGGTG |
| *CBTS6* | XM_016655305.1 | CGGGAGCCAATGAATGGGAA | CAGGCCAGTGACTCCTCAAT |
| *CBTS7* | [XM_016629581.1](https://www.ncbi.nlm.nih.gov/nuccore/XM_016629581.1) | AGTTCAAGGAAACTCTTACCAATAC | CGCACGACTATATTGAGGCT |
| *CBTS8* | [XM_016594649.1](https://www.ncbi.nlm.nih.gov/nuccore/XM_016594649.1) | AACTCCCACCTTGCTCTTGG | GCTTCGTGGCAGTCTAGGAG |
| *CBTS9* | [XM_016631693.1](https://www.ncbi.nlm.nih.gov/nuccore/XM_016631693.1) | CCAAAGCGAGCTTTACGAGC | TCTGCATCGCATCCGTGAAA |

**Table S2.** Primers for semi-quantitative RT-PCR

| **Gene** | **NCBI Accession** | **Primer Forward(5’-3’)** | **Primer Reverse(5’-3’)** |
| --- | --- | --- | --- |
| *CBTS1* | XM_016609836.1 | ACGAGCTAACAAGCTGGTGG | CGCATCCGTGAAAAGCACAA |
| *CBTS2* | XM_016609052.1 | ATGAGTGCAGCAATGGTTGATA | CTTGAACACATCAGAAGACA |
| *CBTS3* | [XM_016582085.1](https://www.ncbi.nlm.nih.gov/nuccore/XM_016582085.1) | ATGAAAGAATTTGGAGGTTC | TCTCAAACTACATTATTTTGCTCCA |
| *CBTS4* | XM_016637837.1 | ACCAACTCGTCGGTCTGTAG | TAACCCCAACATGTGCTGCT |
| *CBTS5* | [XM_016603386.1](https://www.ncbi.nlm.nih.gov/nuccore/XM_016603386.1) | ATGTTGGTAGAAACTCCAGA | TTCATAAATGTATATGTATT |
| *CBTS6* | XM_016655305.1 | AACGGCGGTGTGACAATCAT | TCCTTTGTCGTCAGCGAACT |
| *CBTS7* | [XM_016629581.1](https://www.ncbi.nlm.nih.gov/nuccore/XM_016629581.1) | ATGGAAAGTTCAAGGAAACT | TTCATCATAAGTTGCATAAG |
| *CBTS8* | [XM_016594649.1](https://www.ncbi.nlm.nih.gov/nuccore/XM_016594649.1) | AACTCCCACCTTGCTCTTGG | TGATTCAAAATTTGCCACGT |
| *CBTS9* | [XM_016631693.1](https://www.ncbi.nlm.nih.gov/nuccore/XM_016631693.1) | GGAGTAATAAGCAGATTAAA | TCATATTTTCACGGAGTCAA |

**Table S3.** Primers for vector construction

| **Gene** | **NCBI Number** | **Primer Forward(5’-3’)** | **Primer Reverse(5’-3’)** |
| --- | --- | --- | --- |
| *CBTS1* | XM_016609836.1 | TCCAAGCTTTGCAAAGATGAATCGAGCAATGGATCT | TCGATGCCCACCCTCTTCGAATCTGTCGACATATGA |
| *CBTS2* | XM_016609052.1 | TCCAAGCTTTGCAAAGATGGTTTCTTTTTCAATTA | TCGATGCCCACCCTCTCTTGTAAGCTCGCCAAGCTC |
| *CBTS3* | [XM_016582085.1](https://www.ncbi.nlm.nih.gov/nuccore/XM_016582085.1) | TCCAAGCTTTGCAAAGATGATGGATGATAATACTTCCAG | TCGATGCCCACCCTCTTCAAACATTGACAGGCTCAA |
| *CBTS4* | XM_016637837.1 | TCCAAGCTTTGCAAAGATGGAGGTTAACAATATTGT | TCGATGCCCACCCTCTTCGAACCATTTAAAACATAG |
| *CBTS5* | [XM_016603386.1](https://www.ncbi.nlm.nih.gov/nuccore/XM_016603386.1) | TCCAAGCTTTGCAAAGATGATGAAGCGAGCAATGAATCC | TCGATGCCCACCCTCTTTATATGTCGATAGATTCGA |
| *CBTS6* | XM_016655305.1 | TCCAAGCTTTGCAAAGATGGATAGGAGTTCTGGAGA | TCGATGCCCACCCTCTTTGATCCCATTACAATATGA |
| *CBTS7* | [XM_016629581.1](https://www.ncbi.nlm.nih.gov/nuccore/XM_016629581.1) | TCCAAGCTTTGCAAAGATGGAAAGTTCAAGGAAACTCTTAC | TCGATGCCCACCCTCTTCAAACATTGACAGGTTCAACAAAC |
| *CBTS8* | [XM_016594649.1](https://www.ncbi.nlm.nih.gov/nuccore/XM_016594649.1) | TCCAAGCTTTGCAAAGATGATGTACAAAAATTTAGATGT | TCGATGCCCACCCTCTATATTTTGATAGGTTCAACC |
| *CBTS9* | [XM_016631693.1](https://www.ncbi.nlm.nih.gov/nuccore/XM_016631693.1) | TCCAAGCTTTGCAAAGATGATGAAACAAGATGTGTTCAA | TCGATGCCCACCCTCTTCATATTTTCACGGAGTCAA |

**Table S4.** Gradient mobile phase for UPLC assay

| **Number** | **Time (min)** | **Flow rate (mL/min)** | **Acetonitrile (%)** | **ddH_2_O (%)** |
| --- | --- | --- | --- | --- |
| 1 | Initiation | 0.3 | 20 | 80 |
| 2 | 2 | 0.3 | 40 | 60 |
| 3 | 4 | 0.3 | 60 | 40 |
| 4 | 6 | 0.3 | 80 | 20 |
| 5 | 8 | 0.3 | 100 | 0 |
| 6 | 9 | 0.3 | 100 | 0 |
| 7 | 11 | 0.3 | 80 | 20 |
| 8 | 13 | 0.3 | 60 | 40 |
| 9 | 14 | 0.3 | 40 | 60 |
| 10 | 16 | 0.3 | 20 | 80 |
